# Cognitive decline and psychotropic drugs use in elderly people with mental disorders

**DOI:** 10.1192/j.eurpsy.2021.1121

**Published:** 2021-08-13

**Authors:** K. Cestari De Oliveira, P. Haidamus De Oliveira Bastos

**Affiliations:** 1 Programa De Pós Graduação Em Saúde E Desenvolvimento Na Região Centro-oeste, Universidade Federal de Mato Grosso do Sul, CAMPO GRANDE, Brazil; 2 Programa De Pós Graduação Em Saúde E Desenvolvimento Na Região Centro-oeste, Universidade Federal de Mato Grosso do Sul, Campo Grande, Brazil

**Keywords:** Cognitive decline, Psychotropic drugs, Elderly people, Mental disorders

## Abstract

**Introduction:**

The growth in the number of aged people in the population is considered a worldwide phenomenon, with direct consequences in health systems. The literature indicates an increase in the diagnosis of mental disorders and the use of psychotropic drugs for that population, as well as frequent complaints regarding to cognition.

**Objectives:**

To analyze the possible relationship between cognitive decline and use of psychiatric drugs in elderly with mental disorders, assisted by psychiatric outpatient clinics, city of Campo Grande, state of Mato Grosso do Sul, Brazil.

**Methods:**

Quantitative, exploratory, descriptive and cross-sectional research, with 59 participants.Sociodemographic and clinical variables were collected through semi-structured clinical interviews and medical records. To screen for cognitive decline, the Mini Mental State Examination was used.

**Results:**

Majority of females, with a mean age of 66.75 ± 0.63 years, married, up to 8 years of completed studies and living with family members. The prevalence of depressive disorders was higher (52.54%), with selective serotonin reuptake inhibitor antidepressant use in 67.8%. Most were using 2 or more psychotropics the most prevalent combination being benzodiazepines and antidepressants. 52.5% of the elderly reported cognitive complaints and 45.8% presented Mini Mental scores, suggesting cognitive decline. It was associated with depressive disorders and the consumption of 2 or more psychotropics.

**Conclusions:**

Although there is evidence that psychotropic drugs represent effective strategies for the treatment of mental disorders, the use for this group of elderly should be carefully analyzed, due to the predisposition or worsening of cognitive decline, with impairment to the quality of life of this population.

